# Serum 17*β*-estradiol inversely correlates with circulating group 2 innate lymphoid cells in a cohort of asthmatic patients

**DOI:** 10.3389/fimmu.2025.1555228

**Published:** 2025-08-01

**Authors:** Elizabeth J. Myers, Samuel E. Aamodt, Thomas P. Huecksteadt, Robert Paine, Mustafa Mir-Kasimov, Christopher A. Reilly, Sean J. Callahan, Karl A. Sanders, Kristi J. Warren

**Affiliations:** ^1^ Research Service, Salt Lake City VA Medical Center, Salt Lake City, UT, United States; ^2^ Department of Neurology, University of Utah, Salt Lake City, UT, United States; ^3^ Department of Internal Medicine – Pulmonary Division, School of Medicine, University of Utah, Salt Lake City, UT, United States; ^4^ Division of Human Toxicology, College of Pharmacy, University of Utah, Salt Lake City, UT, United States

**Keywords:** asthma, estrogen, progesterone, neutrophils, eosinophils

## Abstract

**Introduction:**

Asthma is a chronic airway inflammatory disorder that demonstrates a strong clinical bias in females of reproductive age. In this study we evaluated group 2 innate lymphoid cells (ILC2) that play a now well-defined role in allergy and asthma. ILC2 are rare immune cells that demonstrate a strong activation bias in females compared to males in both mice and humans. We hypothesized that ILC2 would be highly activated in people with asthma as compared to healthy, sex-matched controls.

**Methods:**

Subjects with asthma were identified by medical records searching and confirmed through pre-clinic interviews regarding asthma diagnosis. Additional demographic and clinical data were collected from study questionnaires or retrospective chart review. Correlations were determined between immune activation and hormone levels for each study participant regardless of healthy or asthma status.

**Results:**

Results showed that within the asthma groups, female Veterans had higher circulating blood neutrophils compared to males, and males had higher eosinophils compared to females by complete blood cell count. ILC2 trended upwards in male Veterans with asthma compared to female Veterans with asthma (p = 0.086). Females with asthma had a marked reduction in CRTH2+ ILC2 in comparison to healthy female controls. The numbers of ILC2 in correlation to ovarian hormones were determined to show a significant inverse correlation with estrogen levels and ILC2 suggesting that estrogen may suppress ILC2 abundance in circulation.

**Conclusions:**

Additional studies are necessary to determine whether this estrogen-effect extends to the lung and airways of people with asthma.

## Introduction

Asthma is a chronic airway condition that afflicts approximately 350 million individuals worldwide (1). In the United States 4-5% of males and 7-8% of females are diagnosed with asthma (2). Even with extensive efforts to reduce asthma rates there are significant challenges that remain for diagnosing and treating this disease (3). Diet, obesity, childhood versus adult onset, levels and types of pollution, and environmental changes effected by temperatures, allergen levels, and even genetic polymorphisms within the unique host, have complicated the ever-changing landscape of asthmatic airway disease (4). Biological sex is one of the critical variables that influences the development and severity of asthma across the human population (5). The current study was designed to evaluate asthma in male and female US Veterans who experience inhalation exposure to burn pit emissions, toxicants, pollutants of various origins, and agricultural dusts leading to new asthma diagnosis after deployments (6). These new exposures trigger unique airway and respiratory tract inflammatory responses for these deployed individuals; most commonly asthma is reported for these military personnel post-deployment (7). Female Veterans in particular return with higher rates of asthma than do male Veterans indicating that the sex bias seen in civilians with asthma extends into the Veteran population (8, 9). As with other respiratory diseases, sex hormones play a role in this clinical observation (9, 10), and we are the first to characterize the immunobiology behind male versus female asthma in Veterans.

Group 2 innate lymphoid cells, or ILC2, are innate immune cells that demonstrate a stronger activation in females compared to males (11). ILC2 can be activated by the epithelial cell derived cytokine, IL-33. IL-33 is of the family of cytokines called the ‘alarmins’, and like IL-33, TSLP and IL-25 are sentinel cytokines constitutively expressed in airway tissues that are released under conditions of airway stress (12, 13). These alarmins are released during viral infections, cigarette smoke exposures, and pollution exposures (14–16). Once IL-33 ligates its cognate receptor (ST2) on ILC2 it causes a rapid increase of IL-5 and IL-13 production and secretion by ILC2 in the proximal airways. IL-5 and IL-13 support eosinophil survival and recruitment, and program type 2 T helper cell responses in allergy and asthma (17). IL-13 also drives the production of mucus by goblet cells in the airway, and programs the IgE response in B cells (18, 19). Although anti-IL-5 and anti-IL-13 biologics already exist, they have met with limited success for subsets of patients (20). More recently, clinical therapies are under development for neutralizing IL-33 and the IL-33 receptor subunit, ST2, in patients with uncontrolled and severe asthma and in atopic dermatitis (21). To date, these biologic therapies are proving effective in clinical trials, but more studies are needed to fully elucidate the effects of IL-33 as a clinical target.

Ovarian hormones have been proposed for decades as the mechanism by which females develop asthma after puberty (22, 23). One of the most cited statistics for the sex bias in asthma is that 30-40% of women with asthma have perimenstrual worsening of their symptoms (11, 24), and the same statistic applies to pregnant women, with ~40% of those women experiencing worsening of their asthma symptoms during pregnancy (25, 26). Hormonal fluctuations are thought to influence asthma onset and exacerbations in females while testosterone has been shown to attenuate asthma especially in biologically male children with asthma as they reach sexual maturity (27, 28). Mechanistic studies have shown that the androgen receptor on group 2 innate lymphoid cells (ILC2) significantly reduces type 2 inflammatory markers by ILC2. Similarly, we have shown that ILC2 and eosinophil abundance are reduced in experimental asthma when plasma estrogen concentration was consistent and comparable to levels of the early to mid-follicular phase of the menstrual cycle. Those studies did not determine which estrogen receptor(s) was responsible for the suppressive effect by estrogen. There are three known estrogen receptors expressed in mammalian tissues, two with genomic effects (ER*α*, ER*β*), and one with cytoplasmic signaling effects (GPER-1); it is plausible that estrogen may suppress ILC2 responses through at least one of these receptors. The genomic estrogen receptors were recently identified on human blood ILC2 (29). ER*α* has been investigated in depth in various immune cells using ER*α* knockout animals (30), and ER*β* was manipulated pharmacologically in a mixed allergen exposure model to limit pulmonary hyperresponsiveness to methacholine challenge (31). GPER-1 was recently shown to reduce allergic inflammation in mice (32), but to date, it has not been determined whether GPER-1 is expressed on ILC2 or whether ILC2 abundance or activity can be modulated through GPER-1 activation or blockade.

Precision medicine has become routine in the care of patients with asthma, leading us to evaluate whether estrogenic compounds may be effective targets for modulating asthma onset and exacerbations through hormone receptors on human ILC2. In this manuscript we extend our previous work to include an investigation of ILC2 in a clinical cohort of male and female Veterans with and without asthma. Our hypothesis was that estrogen, acting through GPER-1 would significantly reduce cytokine responses in ILC2 isolated from male and female Veterans with asthma. Here we used the GPER-1 agonist, G1, in ex vivo ILC2 activation assays, to show that activating GPER-1 reduced expression of asthma promoting cytokines from circulating ILC2.

## Materials and methods

### Human subjects

Male and female US Veterans with and without asthma were recruited from the greater Salt Lake City area from January 2023 – January 2024. Patient characteristics are reported in **Table 1**. Participants were 26–54 years of age [mean age of cohort = 43.5] and had completed at least a 3–4 year tour of active duty with the United States Armed Services. Healthy controls were identified through advertisements. People with asthma were screened and recruited based on physician diagnosed asthma, and where applicable, pulmonary function tests were examined to confirm asthma status. All participants had not used combustible or electronic cigarettes for at least 1 year prior to enrollment and were excluded from the study if they had additional pulmonary or cardiovascular conditions, such as pulmonary hypertension or COPD. Female participants were excluded if pregnant, lactating, or past reproductive age (i.e. menopausal). In rare cases, female participants were excluded following oophorectomy; but were included if only a hysterectomy was performed. All participants had to be free from respiratory infection and had not used systemic steroids for at least 1 week prior to blood draw. Contraceptive use and prescription use were also recorded in **Table 1**. All study procedures were approved by the R&D committee at the Salt Lake City VA Medical Center and the Institutional Review Board at the University of Utah (IRB# 00120600).

**Table 1:** Demographics of study participants.

	Female asthma	Female healthy	Male asthma	Male healthy	P-value
n/total (%)	16/62 (26%)	16/62 (26%)	14/62 (22%)	16/62 (26%)	n.d.
Years w/asthma [range] SEM	14.82 [0.5-40]4.56	n/a	10.4 [0.5-28] 3.21	n/a	n.d.
*> 19 y.o.*	10/16 (63%)	n/a	8/14 (57%)	n/a	n.d.
*< 19 y.o.*	2/16	n/a	1/14	n/a	n.d.
*Not recorded*	4/16	n/a	4/14	n/a	n.d.
Allergic Rhinitis	15/16	6/16	11/14	4/16	p <0.001
Age [Range] SEM	44.5 [26-54] 8.8	43.7 [35-54] 6.9	44.3 [27-54] 8.1	41.1 [31-53] 7.8	n.d.
BMI [Range] SEM	32.29 [24-44] 1.5	31.28 [23-44] 1.7	35.10 [25-53] 2.0	32.92 [24-48] 2.1	n.d.
VA PFT available	6/16	3/16	9/14	3/16	p = 0.05
Deployments					
*Never*	4/16	n.r.	2/14	n.r.	n.d.
*Yes*	7/16	n.r.	7/14	n.r.	n.d.
*Not recorded*	5/16	n.r.	5/14	n.r.	n.d.
Deployment Region					
*Middle East*	10/16	n.r.	10/14	n.r.	n.d.
*East Asia*	3/16	n.r.	1/14	n.r.	n.d.
*Europe*	3/16	n.r.	3/14	n.r.	n.d.
*Africa*	3/16	n.r.	0/14	n.r.	n.d.
*South Asia*	1/16	n.r.	0/14	n.r.	n.d.
Medications					
*anti-histamines*	11/16	5/16	7/14	2/16	p = 0.01
*LTRAs*	9/16	0/16	3/14	0/16	p < 0.001
*LABA*	9/16	0/16	4/14	0/16	p < 0.001
*SABA*	15/16	0/16	12/14	0/16	p < 0.001
*ICS*	12/16	3/16	9/14	2/16	p < 0.001
Hospital/ER visit for asthma ever	8/16	n/a	3/14	n/a	p = 0.11
Asthma endotype					
*High type 2; eos*	2/16	n/a	4/14	n/a	n.d.
*low type 2; PMN*	8/16	n/a	2/14	n/a	p = 0.04
*Mixed granulocytic*	5/16	n/a	8/14	n/a	p = 0.16
*Paucigranulocytic*	1/16	n/a	0/14	n/a	n.d.
Contraceptives					
*Oral contraceptives*	1/16	n.r.	n/a	n/a	
*IUD*	1/16	n.r.	n/a	n/a	
*Injectable*	1/16	n.r.	n/a	n/a	
*do not use*	7/16	n.r.	n/a	n/a	
*not recorded*	6/16	n.r.	n/a	n/a	

n.d. indicates that between groups comparisons by student t-tests (for 2 groups) or One-Way ANOVA (> 2 groups) were not statistically different P-values are reported when a statistical difference was identified. n.r. indicates the data were ‘not recorded’ and n/a indicates that the results are not applicable to the group.

### Blood collection, tissue processing and sample storage

Up to 100 milliliters (mL) of whole blood was collected from each study participant by the Pulmonary Clinical Research Group at the University of Utah. 8–10 mL of whole blood were collected for serum, 8–10 mL for plasma processing, and up to 80 mL of EDTA treated whole blood for PBMC isolation. Serum and plasma tubes were centrifuged at room temperature for 15 minutes at 900 x g, then aliquoted into 0.65 mL tubes for long-term storage at -80^°^C. For PBMC isolation, whole blood was diluted 1:1 with 1X Dulbecco’s PBS (Fisher Scientific, Waltham, MA, catalog #21031) and gently mixed by inversion 2–3 times. 20 mL of diluted blood was slowly poured into a 50 mL SepMate tube (Stemcell Technologies, Vancouver, Canada, catalog number #85450) containing 20 mL of Lymphoprep to create the density gradient medium (Stemcell Technologies, Vancouver, Canada, catalog #07851). SepMate tubes were centrifuged at 1200 x g for 10 minutes at room temperature. Separated cells were poured into a new 50 mL tube and washed with EasySep buffer (StemCell Technologies, Vancouver, Canada, catalog #20144) and centrifuged at 300 x g for 5 minutes at room temperature. Cells were counted on the TC-20 cell counter (Biorad, Hercules, CA) with trypan blue exclusion to determine viability. Cells were resuspended at a concentration of 20–40 x 10^6^ cells/mL in cryopreservation media composed of 35% Dulbecco’s minimum essential media (DMEM; Fisher Scientific Invitrogen, Waltham, MA), 55% charcoal-scrubbed fetal bovine serum (FBS; Fisher Scientific Invitrogen, Waltham, MA) and 10% DMSO (dimethyl sulfoxide; ATCC, Manassas, VA). 1–2 mL aliquots of PBMC were placed in cryopreservation tubes and stored at -80°for short-term storage (<6 months) and -150°C for long-term storage (>6 months).

### ILC2 enrichment and activation assays

ILC2 were enriched from cryopreserved participant PBMC by negative selection using the human ILC2 enrichment kit from StemCell Technologies (Cat# 17972) according to the manufacturer’s protocol. ILC2 numbers post-enrichment and viability were determined by trypan blue exclusion prior to culture with human recombinant IL-2 (10 ng/mL) (PeproTech Thermofisher, Cat#200-02-10UG), IL-7 (10 ng/mL) (PeproTech Thermofisher, Cat#200-07-10UG) and IL-33 (10 ng/mL) (PeproTech Thermofisher, Cat#200-33-10UG) in RPMI supplemented with antibiotics and charcoal-scrubbed FBS. Estrogen (Sigma, St. Louis, MO) was initially tested at a titrating dose of 1 to 100 pg/mL to confirm regulation of ILC2 at a rate of 10 pg/mL as in our previously published work (33). In the G1 experiments, G1 (Tocris Biosciences, Bristol, UK, #3577) was titrated to a working range of up to 3.2 uM concentration in culture. Viability was determined in every culture experiment to confirm that concentrations of cytokines, hormones and hormone receptor agonists did not significantly reduced cell viability. ILC2 culture was performed in a 96-well, U bottom plate (Corning, Corning, NY) for up to 6 days at 37°C with 5% CO_2_.

### Flow cytometry and Intracellular staining procedures

PBMC from each donor were stained for a panel of markers (**Supplementary Table 1**) to determine CD3+ T cells, CD19+ B cells, PMN, eosinophils, monocytes and group 2 innate lymphoid cells (ILC2) Group 2 innate lymphoid cells were defined as CD45+LIN-[CD3-CD4-CD8-CD19-ter-1-CD15-CD14-CD16- FceR1-CD11b-CD11c] CD127+CRTH2+. For intracellular staining 96-well U bottom plates were removed from the incubator and centrifuged at 300 x g for 5 minutes at room temperature. Supernatants were collected and stored at –80 ° C until confirmatory ELISAs were performed. Cell pellets were resuspended in RPMI with GolgiStop (BD Biosciences, San Diego, CA, #554724) for 2–4 hours at 37° C. Cells were stained extracellularly in 50–100 uL of FACS staining buffer with BSA (BD Pharmingen; San Diego, CA) and supplemented with antibody cocktail for 20 minutes. Following staining, cells were washed and fixed with 1x FoxP3 Fixation buffer following manufactures instructions. Permeabilization buffer (Fisher Scientific, Waltham, MA #00-5523-00) was added before staining with intracellular antibody cocktail for 20 minutes. After staining the samples were washed twice prior to data acquisition using a Cytek Aurora spectral cytometer (Cytek Biosciences, Fremont, CA). Gating schematics and cellular data analysis was performed using FlowJo v. 10 software.

### ELISA

Hormones were extracted from 250 uL of serum acquired from each donor using the method adapted from Reilly et al. (2004) (34). 17*β* estradiol (Enzo, Farmingdale, NY cat# ADI-900-174) and progesterone (Enzo, Farmingdale, NY Cat# ADI-900-011) were measured in extracted serum according to manufacturer’s instructions. 100 uL of plasma was added to each of the following ELISA plates after a 2-fold dilution was performed using reagent diluent that accompanies the Ancillary kit 2 (R&D Systems, Minneapolis, MN, #DY008). Plasma was analyzed for human IL-5 (cat# DY205), IL-8 (cat# DY208), IL-10 (cat# DY217), IL-13 (cat# DY213), IL-17 (cat# DY317), Amphiregulin (Areg) (cat# DY262) and IL-33 (cat# DY3626) using the Duoset ELISA kits from R&D Systems. Cell culture supernatants were analyzed for IL-13 using the above listed R&D Duoset.

### Statistical analysis

Data are presented as mean ± standard error of mean (SEM). Statistics were performed using a One-way analysis of variance (ANOVA). A Kruskal-Wallis post-test was used to compare significant differences between independent groups. Simple linear regression was used to compare estrogen levels with ILC2 counts per mL of blood collected and as a percentage of ILC2 per CD45+ cells. A Pearsons correlation matrix was constructed to determine correlates of BMI, with various immune outcomes measured by flow cytometry, and hormones. All statistical tests were completed using GraphPad Prism (version 10). In all analyses, p values less than 0.05 were considered statistically significant.

## Results

### Asthma study population characteristics and neutrophilic versus eosinophilic asthma endotypes in male versus female Veterans with asthma

We initiated these studies to evaluate sex differences in asthma in this unique patient population in comparison to healthy controls. Participant demographics and patient information are reported in **Table 1**. A total of 62 Veterans were recruited, 32 females; 16 with asthma and 16 without asthma, and 30 males were recruited; 14 with asthma and 16 without asthma. Most of the female Veterans had adult-onset asthma (10/16; 63%), whereas 8 of 14 (57%) male Veterans reported developing asthma after 19 years of age. 15 of 16 females with asthma and 11 of 14 males with asthma reported allergic rhinitis in the study questionnaire. There were no differences between mean age and BMI for the four groups; however, most participant BMI exceeded 31 kg/m^2^, including control participants without asthma.

Medical records were examined for pulmonary function testing (PFT) results to better characterize asthma in the patient population. Surprisingly, we found that fewer females with asthma had VA pulmonary function measurements available in comparison to males with asthma (p < 0.05, by student t-test). We further determined that more women were stratified into the neutrophilic, low T2 endotype, based on plasma IL-17 and IL-8, the presence of PMN in circulation by clinic-derived complete blood counts, and flow cytometry measurements made at the time of blood draw (p = 0.039). Subsets of both males and females with asthma were characterized as a mixed granulocytic phenotype due to a moderately high presence of PMN and normal to moderately high eosinophils, higher levels of lymphocytes and the detection of IL-13 and IL-17. Lastly, eosinophilic asthma made up a low number of the asthma endotypes characterized; only 2 of 16 females had above normal circulating eosinophils, and 4 of 14 males with asthma had elevated eosinophils. For these people with eosinophilic asthma, we confirmed that those individuals had no IL-17 in circulation, low levels of IL-8, and a detectable amount of IL-5 and IL-13 in plasma. More females also reported using 4 to 5 drugs to control their asthma and half of the women with asthma reported a hospitalization or emergency room (ER) visit due to an asthma attack in their lifetime. Males reported fewer drugs by questionnaire and only 3 of 14 reported emergency room or hospitalization related to asthma. Questionnaire data was compiled for prescription drugs, oral contraceptives use, and deployments in **Table 1**.

**Figure 1:**
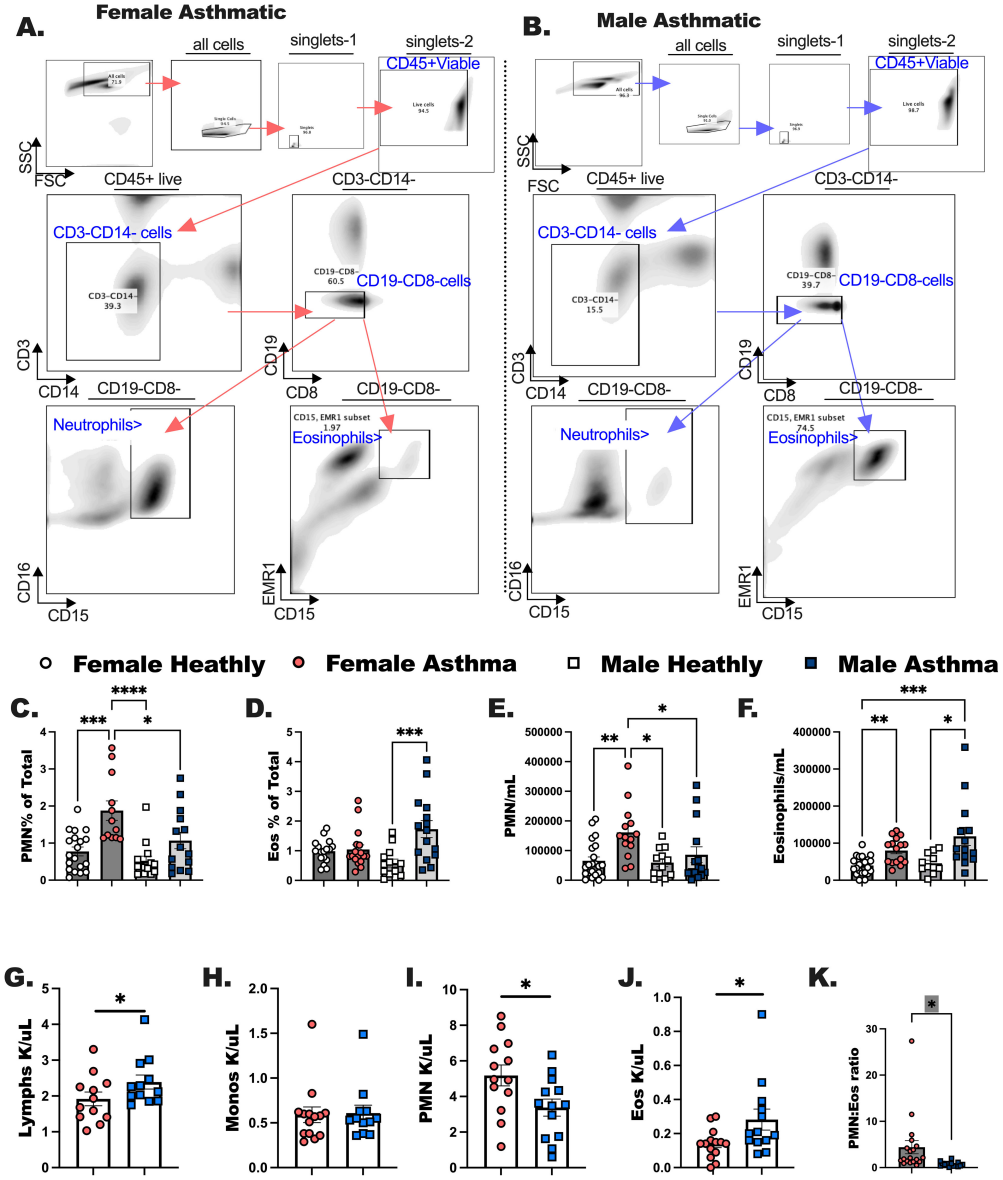
Complete blood counts determined in the clinic are comparable to flow cytometric measurements of immune populations in peripheral blood mononuclear cells (PBMC). Males and females with asthma and healthy Veterans were recruited through the Salt Lake City VA Medical Center for blood collection. Flow cytometry was performed to quantitate both PMN and Eos. **(A, B)** The flow gating strategies are shown for these experiments. **(C, D)** Percentage of neutrophils and eosinophils per total CD45+ cells were determined for each study participant. **(E, F)** The counts for PMN and Eos per milliliter of blood collected from each participant was calculated. **(G-J)** Complete blood cell counts were determined from medical records; **(G)** Lymphocytes, **(H)** monocytes, **(I)** neutrophils (PMN) and **(J)** eosinophils (Eos). **(K)** The ratio of PMN to Eos are shown for female and male asthmatics comparatively. Statistical analysis was performed using a one-way ANOVA with a Kruskal-Wallis post-test. N=30 with asthma, N=32 healthy. Statistical significance is shown as * between groups p <0.05; ** indicates P < 0.01; *** p < 0.001; **** P < 0.0001.

We performed cell differentials using flow cytometry on whole blood processed into PBMC from each participant (**Figures 1A–F**). In these studies, PMN and eosinophils were statistically higher in individuals with asthma as compared to healthy, sex-matched controls (* indicates a significant asthma effect by one way ANOVA, and Kruskal-Wallis post-test). Complete blood counts (CBC) were taken during clinical visits for males and females with asthma (**Figures 1G–K**). Males with asthma had more lymphocytes and eosinophils compared to females with asthma (**Figures 1G, J**), and females with asthma had more neutrophils compared to the males with asthma (**Figure 1I**). Monocytes (**Figure 1H**) and basophils (data not shown) were not different between males and females with asthma. Complete blood cell counts were used to compile the ratio of neutrophils to eosinophils (**Figure 1K**). In this comparison PMN to Eos ratio was significantly elevated in the females with asthma compared to males with asthma. This might be due to these individuals having eosinophilic asthma or a mixed granulocytic phenotype, as compared to females who tended to have the predominant neutrophilic endotype.

### The dominant lymphoid cell populations are differentially modulated in healthy versus asthma status

**Figure 2:**
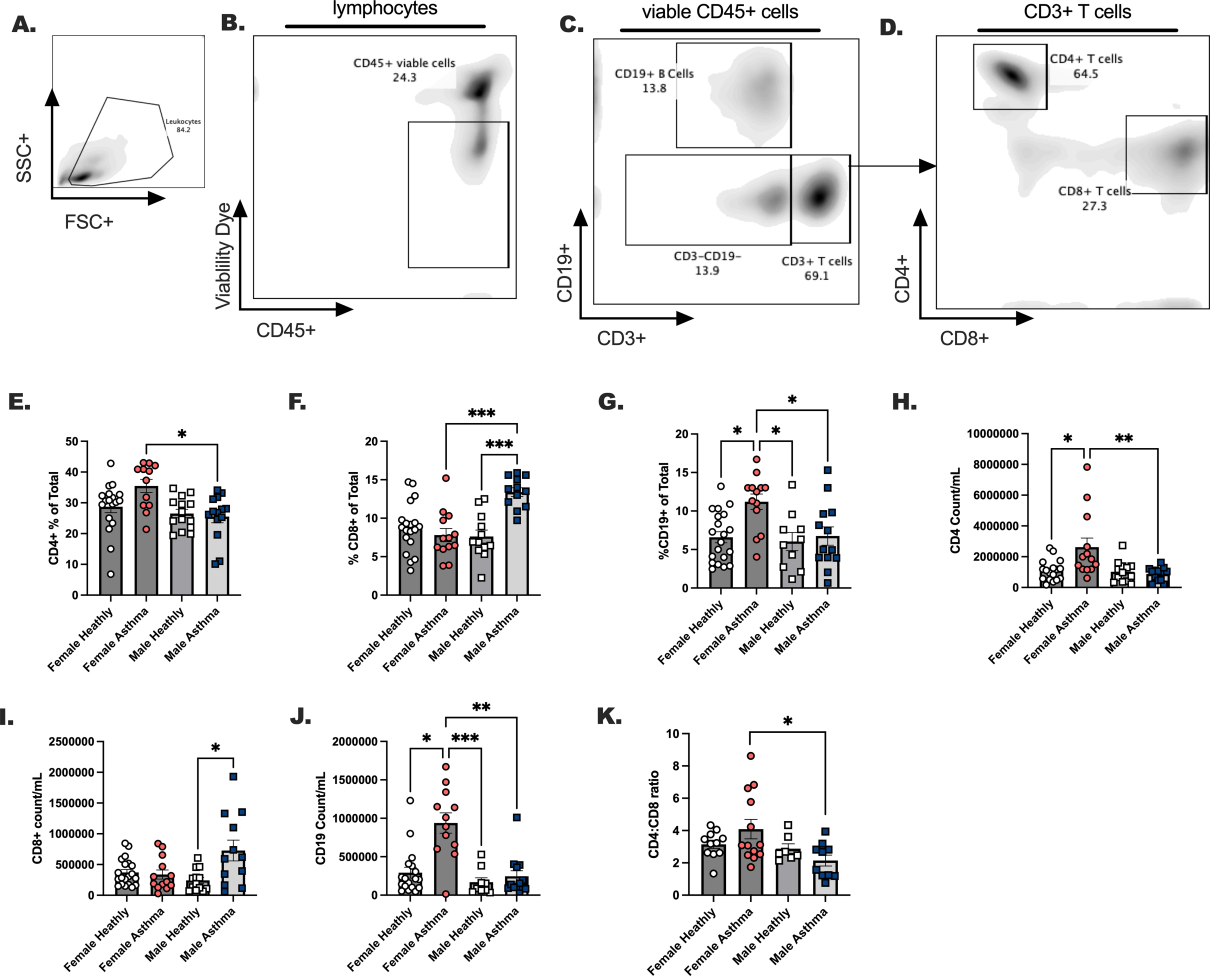
The dominant lymphoid cell populations are differentially modulated in healthy versus asthma status, and in males versus female participants with asthma. Males and females with asthma and healthy veterans were recruited through the Salt Lake City VA Medical Center for blood collection. **(A–D)**. A gating schema is shown for the detection of CD4 and CD8 positive T cells and CD19+ B cells in the blood. Lymphocyte percent of total **(E)**. CD4+ T cells, **(F)** CD8+ T cells **(G)** CD19+ B cells. **(H-K)**. Lymphocyte total counts per milliliter of blood; **(H)** CD4+ T cells, **(I)** CD8+ T cells, and **(J)** CD19+. **(K)**. CD4:CD8 ratios are shown. Flow cytometry was performed to quantitate lymphocytes. Statistical analysis was performed using a One-way ANOVA with a Kruskal-Wallis post-test. N=30 with asthma, N=32 healthy. Statistical differences between groups are shown as * p < 0.05, ** indicates p < 0.01, *** indicates p < 0.001.

We measured the major lymphoid populations comparatively in males and females with asthma compared to healthy, sex-matched controls (**Figures 2A–D**). While we noted no statistical differences in the frequencies and counts per milliliter of blood for total CD3+ T cells (data not shown), we did notice that CD4+ T cells were elevated in females with asthma compared to males (**Figures 2E, H**), while CD8+ T cells were increased in males compared to females with asthma (**Figures 2F, I**). Traditionally, one would anticipate a 3-4:1 ratio of CD4 T cells to CD8 T cells in circulation, but in the case of the males with asthma this ratio was lower in comparison to females with asthma (**Figure 2K**). B cells were elevated in the blood of females with asthma markedly over all other groups (**Figures 2C, G, J**). Taken together, this indicated that cell differentials between male and female Veterans with asthma demonstrate unique immune phenotypes that had previously not been determined for these individuals. We next assessed whether the ILC2 developed differently in these asthmatic versus healthy Veterans.

### ILC2 are decreased in the blood of female Veterans with asthma in comparison to healthy sex-matched controls

**Figure 3:**
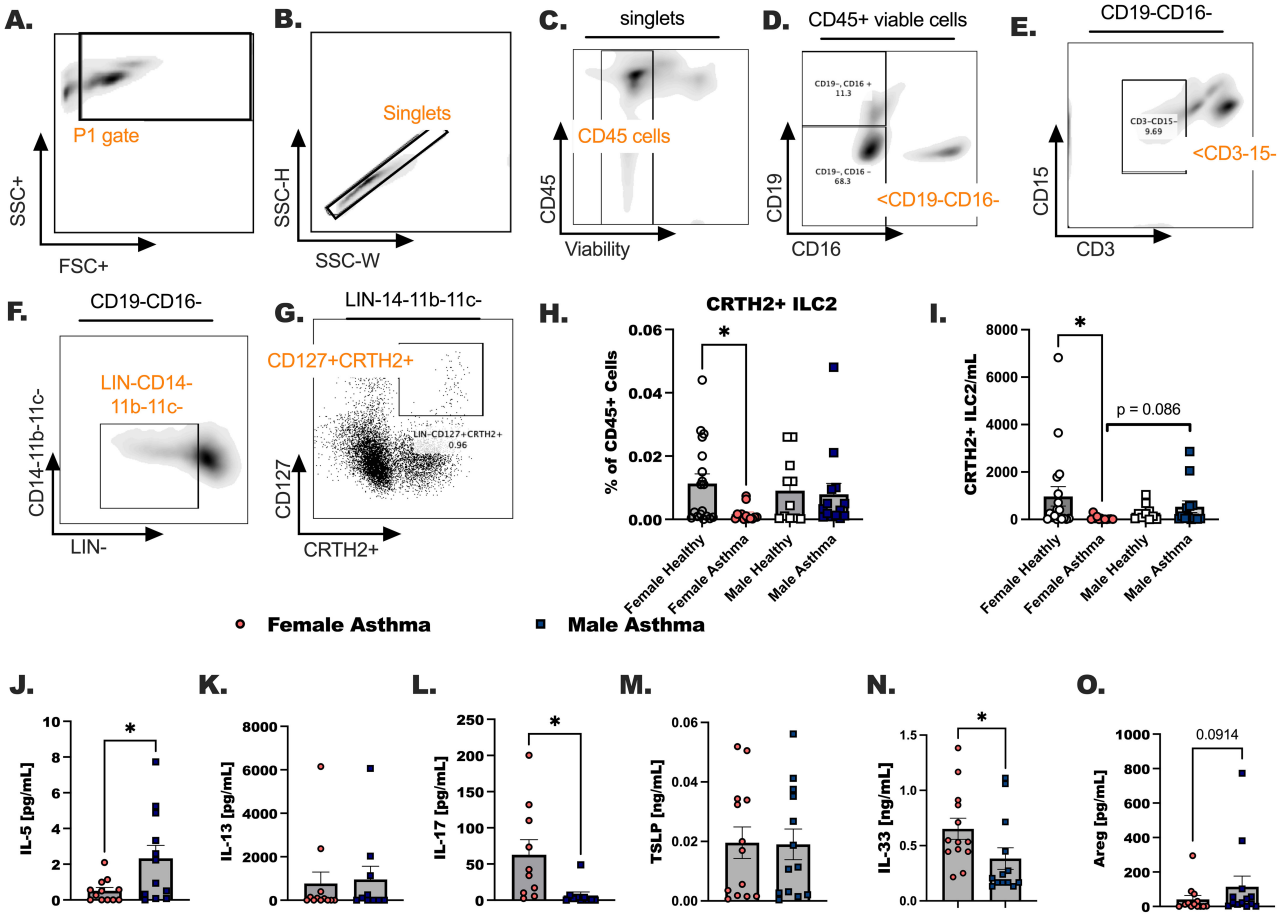
Group 2 innate lymphoid cells are increased in the blood of male and female Veterans with asthma in comparison to healthy sex-matched controls. **(A-G)** A representative flow gating strategy is shown for identifying the CD127+ CRTH2+ ILC2. **(H)**. The percentage of the total viable CD45+ cells that are CRTH2+ ILC2 was determined by flow cytometry. **(I)**. The count of CRTH2+ ILC2 per mL of blood collected are shown. **(J-O)** Cytokine detection in plasma by ELISA. **(J)** IL-5, **(K)** IL-13 **(L)** IL-17 **(M)** TSLP, **(N)** IL-33, and **(O)** Amphiregulin (Areg). Statistical analysis was performed using a One-way ANOVA with a Kruskal-Wallis post-test. N=30 with asthma, N=32 healthy. Statistical difference between groups is shown as * p < 0.05. Exact p-value included for statistical trend between 0.05 and 0.1.

Our previous publications demonstrated ILC2 were increased in female hosts compared to male hosts in experimental asthma (35), leading us to hypothesize that circulating ILC2 would increase in participants with asthma compared to healthy participants. We subsequently anticipated higher numbers of circulating ILC2 in females with asthma compared to males with asthma in the Veteran population. ILC2 were designated as CD45+ lineage negative (LIN-) CD127+CRTH2+ (**Figures 3A–I**). Surprisingly, ILC2 as a percentage of CD45+ cells and ILC2 per milliliter were decreased in females with asthma in comparison to healthy female controls (* p < 0.05). Although in males and females with asthma when ILC2 per mL of blood collected were compared we did detect a trend (p = 0.086) towards a significant sex difference. We additionally measured ILC2-specific cytokines in the asthmatic study populations (**Figures 3J–O**). First, both IL-5 and IL-13 were measured in the plasma of each participant (**Figures 3J, K**). IL-5 was higher in males compared to females with asthma. IL-17 levels were lower when comparing females to males with asthma (**Figure 3L**). The two alarmins, TSLP and IL-33, were measured in the plasma of each participant in the study (**Figures 3M, N**). IL-33 was significantly elevated in females with asthma in comparison to males with asthma, and TSLP levels were not different in the plasma between any of the groups. Lastly, amphiregulin (Areg) was measured and trend upwards in the plasma of male asthmatics compared to female asthmatics (**Figure 3O**). IL-5 and IL-13 are readily secreted from ILC2 once they are activated by IL-33 in our previous studies and by others. However, while both ILC2 specific cytokines were elevated in the participants with asthma compared to healthy individuals, we detected no biological sex differences between males and females with asthma based on the levels of IL-13 detected in plasma.

### Estrogen inversely correlates with the suppression of ILC2 in males and females regardless of healthy or asthma status

**Figure 4:**
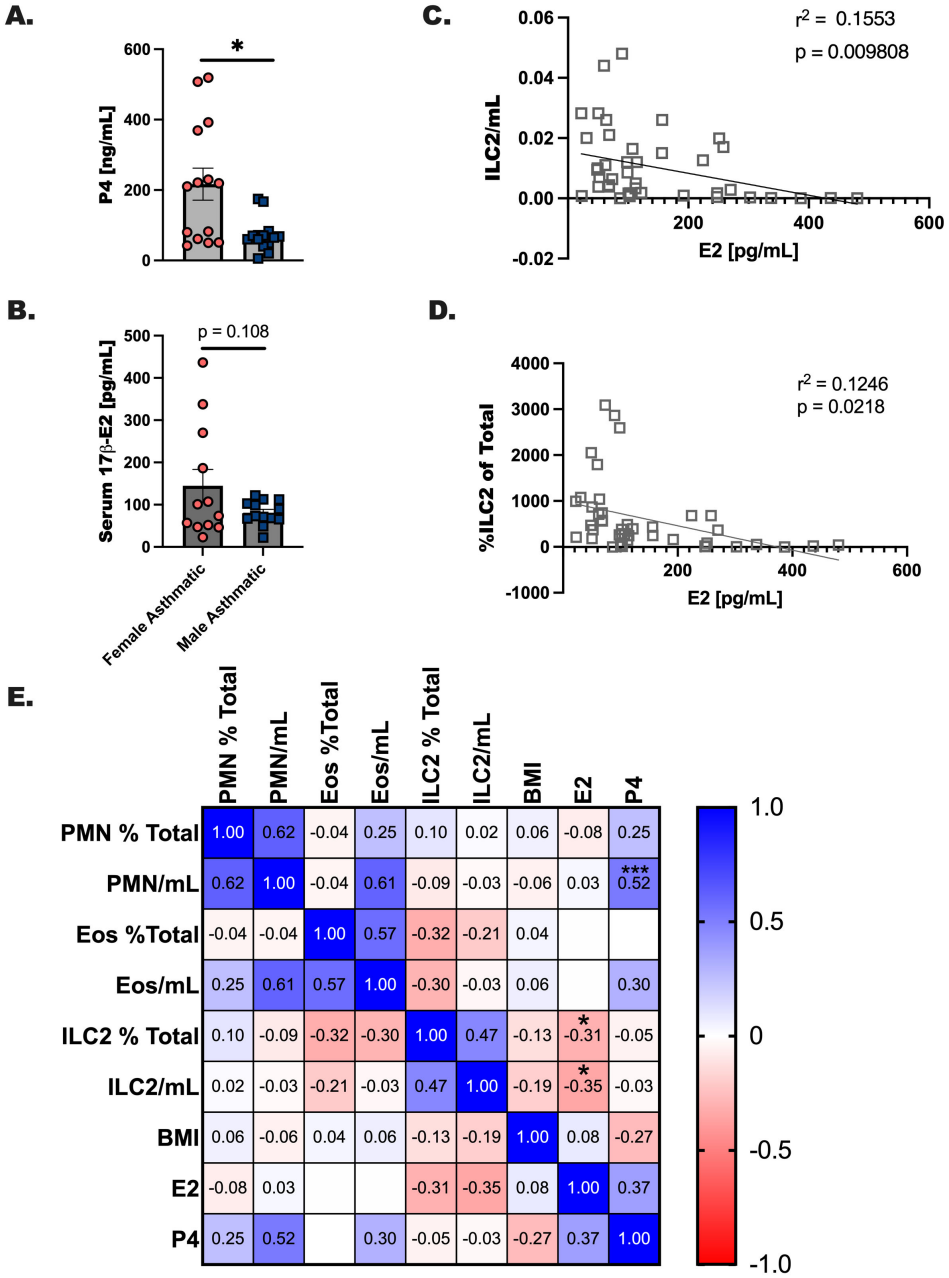
Estrogen inversely correlates with ILC2 numbers in circulation regardless of biologic sex or asthma status. **(A, B)** Serum was processed from whole blood collected from each donor and extracted prior to hormone level determination. **(A)** Progesterone and **(B)** estrogen levels were determined after extraction using ELISA. Donor data was compiled for all study participants, where complete data was available subject data was correlated with **(C)** ILC2/mL of blood collected and **(D)** the % of ILC2 per total CD45+ cells by simple linear regression, and **(E)** a Pearson’s correlation matrix for hormones, BMI and immune populations. * indicates a p-value < 0.05, *** p < 0.001.

In our previous report we noted a significant suppression of eosinophils and ILC2 in an experimental asthma model where 17 *β*-estradiol was delivered at steady levels to ovariectomized female mice compared to sham operated controls prior to allergen challenge. Furthermore, clinical studies have noted that estrogen-based contraceptives are linked to a reduction in asthma onset. In the current study we measured progesterone (**Figure 4A**) and estrogen (**Figure 4B**) in asthma donors including males and females. It has been reported in the literature that visceral adipose tissue is a moderate source of estrogen in overweight individuals. Because we noted higher BMI in all our participants, including males, we were not surprised to measure detectable levels of estrogen in all donors (36). Next, we performed simple linear regression to determine whether there were correlations between ILC2 numbers and estrogen in both male and female participants with asthma (**Figures 4C, D**). Percentage of ILC2 in the blood were inversely correlated with estrogen levels, and when levels of estrogen were compared to the number of ILC2 per mL of blood collected from each study participant. We created a Pearson’s correlation matrix to determine whether interactions are potentially occurring between other innate immune cells including neutrophils (PMN) and eosinophils (Eos), BMI, and ovarian hormones. Using this second statistical test, we detected a similar inverse correlation with estrogen and ILC2 per mL of blood collected (r = -0.35, p < 0.05), and with estrogen and % ILC2 of total CD45+ cells (r = -0.31, p < 0.01) (**Figure 4E**). Progesterone positively correlated with neutrophils in this analysis (r = 0.62, p < 0.05). In summary, these data confirmed that ILC2 are modulated by the concentration of estrogen in the serum at the time of the blood draw.

### GPER-1 is expressed on ILC2 from both healthy people and donors with asthma

**Figure 5:**
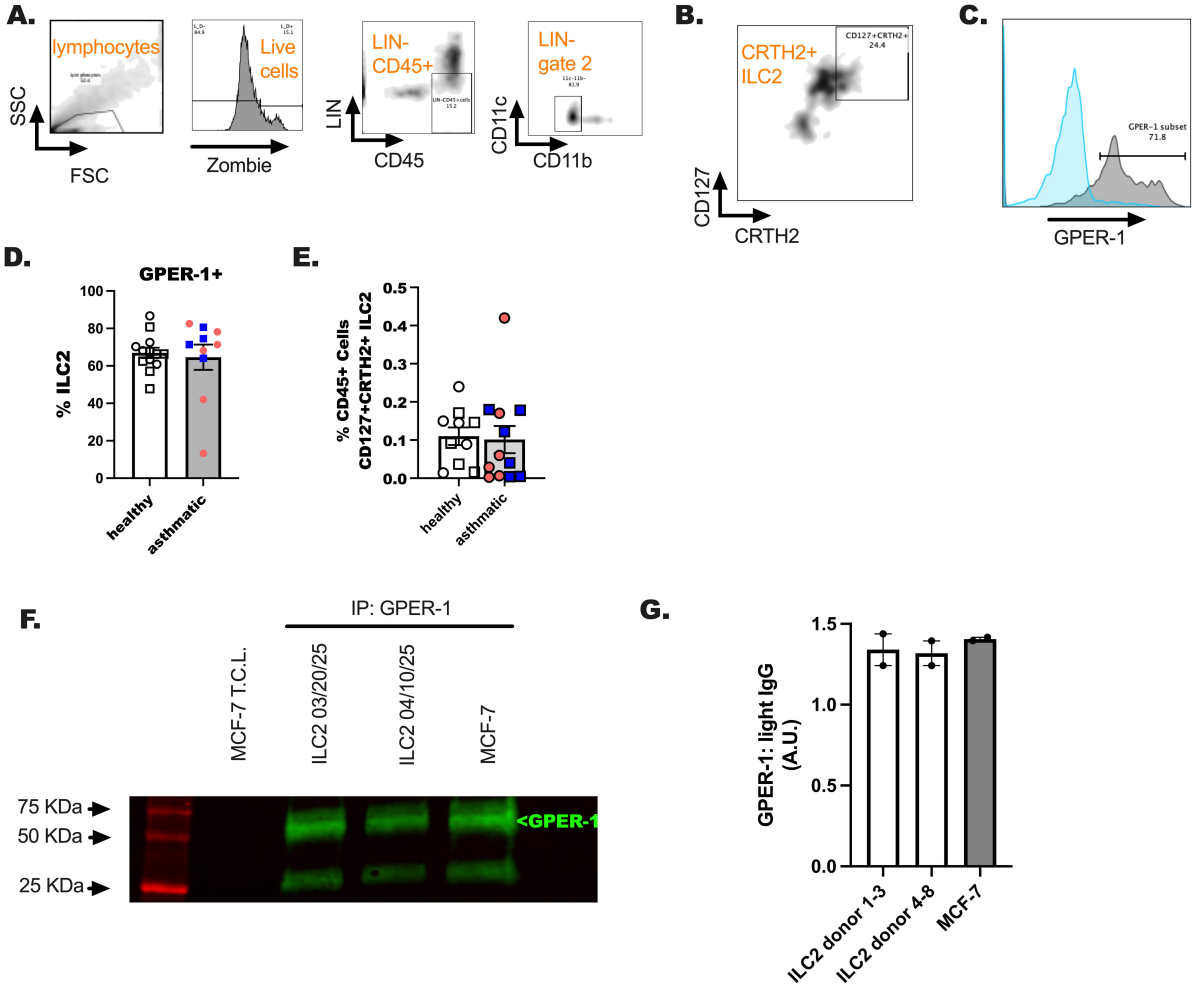
An assessment of GPER-1 on ILC2 of healthy and US Veterans with asthma. Hormone receptor expression was determined on a subset of study participants from all groups. **(A–C)** A representative flow gating schematic was included for reference. **(D, E)**. 1–2 X 10^6^ CD45+ events were collected and gating for ILC2 using LIN- gating and CD11b/c- dump gating to identify ILC2 (CD127+CRTH2+) was performed. **(E)**. The percentage of ILC2 per total cells was determined. **(F, G)** ILC2 were sorted using the schema in **(A, C)** and cultured for 5 days in IL-2, IL-7 and IL-33. Cells were lysed and GPER-1 was immunoprecipitated (5 ug/mL) from the cell lysates (30 ug), followed by probing for GPER-1 (1 ug/mL). MCF-7, ovarian cancers cells, were included as a GPER-1 expressing positive control. Image J software was used to quantitate GPER-1 expression relative to IP light chain antibody. A simple student t-test was used in these studies to compare the asthma and healthy results for these studies. No differences were detected in unpaired analysis; n = 12 for healthy donors and n = 8 for the donors with asthma. An equal number of male and female donors were represented in these studies.

We next investigated whether the G-protein coupled estrogen receptor 1 (GPER-1) was expressed on ILC2 following the discovery that these cells and estrogen were negatively correlated. PBMC were untreated and stained for ILC2 markers and the G protein-coupled estrogen receptor (GPER-1) by extracellular staining methods used previously (**Figures 5A–E**) (37). We showed measurable levels of GPER-1 on blood CRTH2+ ILC2 (**Figures 5C, D**). There were no differences in receptor expression in these studies between healthy participants and participants with asthma. Additional time by treatment interaction studies are needed to fully characterize the fluctuations in ovarian hormones and the levels of this receptor expression on ILC2. We anticipate that hormones may influence immediate reactions in ILC2 based on the results from our next studies. Lastly, we measured GPER-1 in circulating ILC2 by immunoprecipitation and subsequent western blotting (**Figures 5F, G**) (38).

### The GPER-1 agonist, G1, suppresses IL-33 induced ILC2 activation

**Figure 6:**
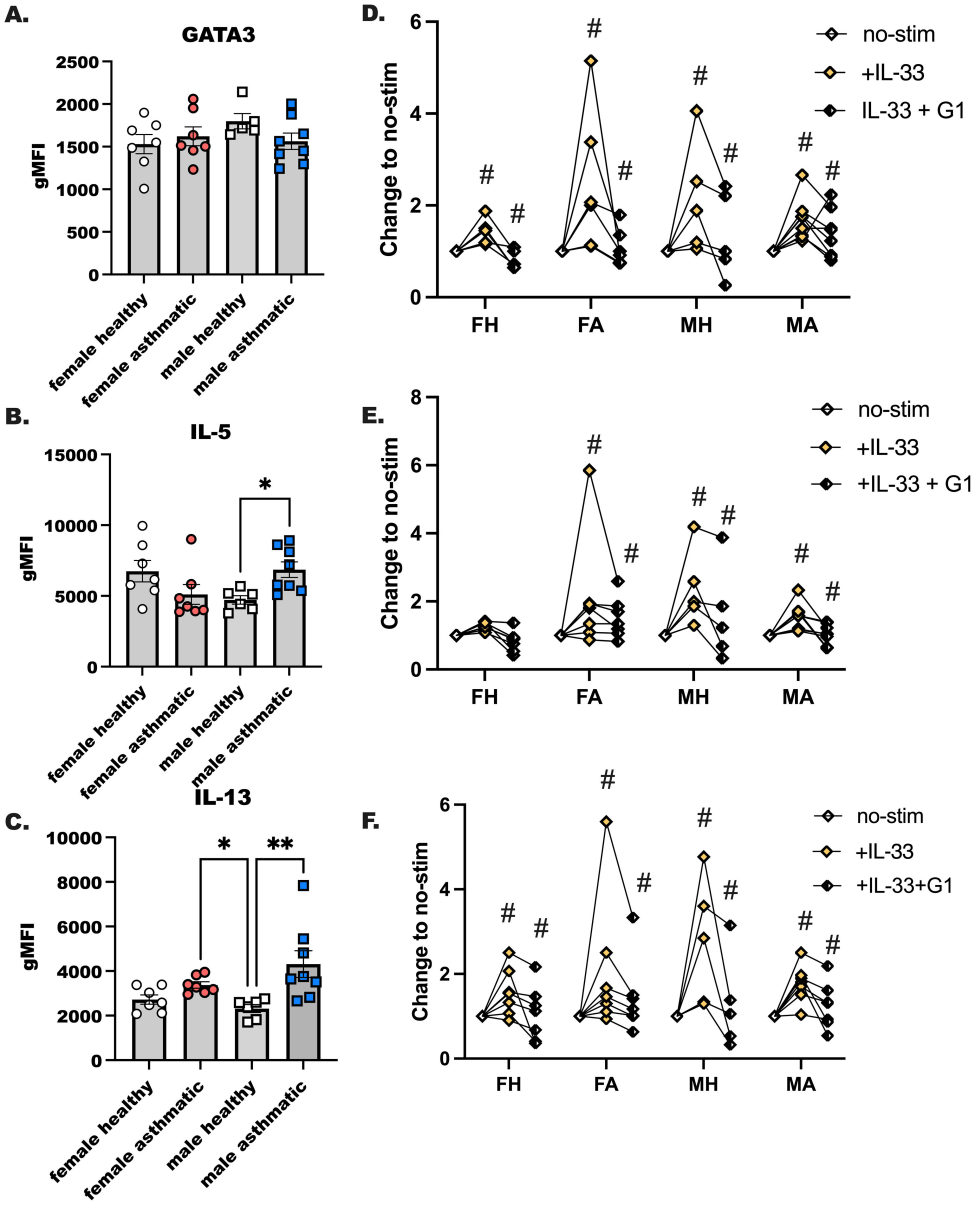
The GPER-1 agonist, G1, suppresses IL-33 induced ILC2 activation. ILC2s were sorted from PBMC of healthy and male and female Veteran participants with asthma. **(A–C)** ILC2s were enriched from PBMC and cultured with maintenance cytokines (IL-2 and IL-7) and the ILC2 activating cytokine IL-33 for 4 days; all cytokines were used at 10–20 ng/mL of media. Flow cytometry was performed with intracellular staining to determine ILC2s as CD45+ LIN- CD127+CRTH2+ cells that express **(A)** GATA3, **(B)** IL-5 and **(C)** IL-13. **(D-F)** ILC2s were enriched as before from PBMC from a second subset of healthy and asthmatic, male and female donors. Cells were cultured as before with maintenance cytokines (IL-2, -7) and IL-33 with and without G1, the GPER-1 agonist. **(D)** GATA3, **(E)** IL-5, and **(F)** IL-13 expression was measured with intracellular flow cytometry. A One-Way ANOVA was used to determine statistical significance, with a Kruskal-Wallis non-parametric test used to determine between group differences. (n= 6-8). For **(D-F)**, a Two-Way ANOVA with repeated measures was used to determine significant effects of IL-33 activation in the various donors, and effects of G1 (n=5-6). # indicates a significant change from no-stim (p < 0.05). * indicates p < 0.05, while ** indicates p < 0.01.

We next assessed the activation potential of ILC2 by IL-33 in our participants after finding that plasma IL-33 was higher in female asthmatics compared to male asthmatics (**Figure 3D**). We have reported that ILC2 are more activated by IL-33 in female mice as compared to male mice, and others have shown more ILC2 in females with severe asthma compared to males with severe asthma. Those studies also showed a higher expression of IL-5 in those female asthmatics compared to male asthmatics. In the current study, however, we did not detect sex differences in IL-5 or IL-13 production by human ILC2 stimulated with IL-33 (**Figures 6B, C**). GATA3 expression in ILC2 was not different when we compared all groups to one another (**Figure 6A**). ILC2 from male asthmatics made more IL-5 and IL-13 when they were stimulated with IL-33 and compared to healthy male control ILC2 (**Figures 6B, C**). Finally, we stimulated ILC2 from each group of donors with IL-2 and IL-7 (no-stim), with IL-33, and with IL-33 and G1 to activate the GPER-1 receptor (**Figures 6D–F**). We noted reduction in GATA3, IL-5, and IL-13 production when we activated GPER-1 in conjunction with IL-33 stimulation in all groups. This shows that ILC2 may be reduced with GPER-1 activation highlighting a previously unknown target for limiting ILC2-driven allergic inflammation.

## Discussion

Sex differences in asthma are well established in the literature, yet, the mechanisms of action of hormones or X-chromosome-linked differences have only been investigated in a relatively small number of studies. For decades asthma has been characterized as a chronic airway condition with eosinophils in the sputum and airways, driven by IL-4, IL-5 and IL-13 from type 2 CD4+ T helper cells. Only recently were ILC2 defined, the importance of the alarmins explored, and the direct association with ILC2, and their IL-5 and IL-13 production, with allergy and asthma determined. The importance of the alarmins, to include TSLP, IL-25 and IL-33, is that they are constitutively expressed by epithelial cells, and once released, activate Th2 cells and ILC2 responses in multiple allergic conditions and asthma. We initiated the current study to characterize sex differences in asthma related to the ovarian hormones in females with asthma with the intent of recruiting males with asthma as controls. Surprisingly, we discovered a statistically significant inverse correlation between ILC2 and serum 17 *β*-estradiol that extended to both sexes. The estrogen effect here fits with our recent publication on stabilizing estrogen levels and limiting ILC2. This certainly is not the only role for estrogen and estrogen receptors in asthma. The estrogen receptor *α* was shown to have no effect on ILC2 directly but did modulate the release of IL-33 from airway cells in a recent publication (39). We activated GPER-1 and found that this limited IL-33-induced cytokine production by human ILC2. Although much more work is required for GPER-1 to be clinically applicable, this at least suggests a novel target for limiting the high type 2 (T2) phenotype or eosinophilic endotype of asthma.

There were a few limitations in our study. The first being that endotypes of asthma were not clearly defined in medical charts. We used a combination of clinical data, with flow cytometry and plasma-detected cytokines (IL-8, IL-17, IL-5 and IL-13) to make our determination of endotypes for each participant. Additionally, we were unable to get complete menstrual cycle data from the female participants. Only a small number of female participants who completed the menstrual cycling questionnaire could provide the date of their last menstrual cycle. When comparing the ILC2 numbers with serum concentrations of estrogen it is plausible that ILC2 may fluctuate monthly in females, however, this is speculation at this point, and should be evaluated further. Hypothetically, circulating estrogen may modulate the pool of ILC2 available in circulation during an exacerbation. Next, circulating ILC2 are different than a lung resident ILC2, and future work should investigate whether hormonal changes in females modulate airway or sputum derived ILC2. Tissue IL-33 secretion during an allergen challenge or exacerbation may induce the influx of ILC2 into the lungs (40), but whether there are post-translational modifications of IL-33 and secreted ST2 that may be involved in the bioavailability of IL-33 to ILC2 is not known either. To add to the complexity is that androgens and ovarian hormones may modify all these layers of IL-33 biology.

Progesterone also merits an examination given how closely progesterone tracked with neutrophil levels. Because this hormone, with estrogen, is produced then drops leading into menstrual cycling it is likely that women with perimenstrual worsening may be the best clinical population in which to study progesterone and ILC2. The relationship with ILC2 and neutrophils may be related to chemokine expression. Our group published data showing an effect of P4 in amplifying ILC2 responses, and even performed ex vivo culture of P4 only with human ILC2 to show increased chemokine secretion and PR expression. Whether these artificial cell culture systems accurately mimic clinical presentation requires tailored investigations perhaps with female asthmatics with perimenstrual worsening (PMA) compared to those without PMA to explore that hypothesis.

In conclusion, asthma demonstrates a strong bias in females that are of reproductive age. Our data confirm again that people with asthma have elevated numbers and activation of innate lymphoid cells in comparison to healthy. Uniquely, our data also show that female Veterans that are asthmatic have differential expression of cytokines (more IL-33) and our female cohort was more likely to present with neutrophilic asthma as compared to male Veterans. Additional studies are necessary to determine personalized treatment strategies for these women and men with this complicated airway disease.

## Data Availability

The original contributions presented in the study are included in the article/**Supplementary Material**. Further inquiries can be directed to the corresponding author.

## References

[1] Forno E Brandenburg DD Castro-Rodriguez JA Celis-Preciado CA Holguin F Licskai C. Asthma in the Americas: an update: A joint perspective from the Brazilian thoracic society, Canadian thoracic society, latin American thoracic society, and American thoracic society. Ann Am Thorac Society. (2022) 19:525–35. doi: 10.1513/AnnalsATS.202109-1068CME, PMID: 35030062 PMC8996271

[2] Zimmermann N Hogan SP Mishra A Brandt EB Bodette TR Pope SM. Murine eotaxin-2: a constitutive eosinophil chemokine induced by allergen challenge and IL-4 overexpression. J Immunol. (2000) 165:5839–46. doi: 10.4049/jimmunol.165.10.5839, PMID: 11067944

[3] Eggert LE He Z Collins W Lee AS Dhondalay G Jiang SY. Asthma phenotypes, associated comorbidities, and long-term symptoms in COVID-19. Allergy. (2022) 77:173–85. doi: 10.1111/all.14972, PMID: 34080210 PMC8222896

[4] Sharma S Gerber AN Kraft M Wenzel SE. Asthma pathogenesis: phenotypes, therapies, and gaps: summary of the aspen lung conference 2023. Am J Respir Cell Mol Biol. (2024) 71:154–68. doi: 10.1165/rcmb.2024-0082WS, PMID: 38635858 PMC11299090

[5] Miyasaka T Dobashi-Okuyama K Kawakami K Masuda-Suzuki C Takayanagi M Ohno I. Sex plays a multifaceted role in asthma pathogenesis. Biomolecules. (2022) 12(5):650. doi: 10.3390/biom12050650, PMID: 35625578 PMC9138801

[6] Silveyra P Fuentes N Rodriguez Bauza DE. Sex and gender differences in lung disease. Adv Exp Med Biol. (2021) 1304:227–58. doi: 10.1007/978-3-030-68748-9_14, PMID: 34019273 PMC8221458

[7] Savitz DA Woskie SR Bello A Gaither R Gasper J Jiang L. Deployment to military bases with open burn pits and respiratory and cardiovascular disease. JAMA Netw Open. (2024) 7:e247629. doi: 10.1001/jamanetworkopen.2024.7629, PMID: 38662371 PMC11046344

[8] Rivera AC Powell TM Boyko EJ Lee RU Faix DJ Luxton DD. New-onset asthma and combat deployment: findings from the millennium cohort study. Am J Epidemiol. (2018) 187:2136–44. doi: 10.1093/aje/kwy112, PMID: 29893775 PMC6166206

[9] Pugh MJ Jaramillo CA Leung KW Faverio P Fleming N Mortensen E. Increasing prevalence of chronic lung disease in veterans of the wars in Iraq and Afghanistan. Mil Med. (2016) 181:476–81. doi: 10.7205/MILMED-D-15-00035, PMID: 27136656

[10] Barth SK Dursa EK Bossarte R Schneiderman A. Lifetime prevalence of respiratory diseases and exposures among veterans of operation enduring freedom and operation Iraqi freedom veterans: results from the national health study for a new generation of U.S. Veterans. J Occup Environ Med. (2016) 58:1175–80. doi: 10.1097/JOM.0000000000000885, PMID: 27930474 PMC5482227

[11] Zhang GQ Ozuygur Ermis SS Radinger M Bossios A Kankaanranta H Nwaru B. Sex disparities in asthma development and clinical outcomes: implications for treatment strategies. J Asthma Allergy. (2022) 15:231–47. doi: 10.2147/JAA.S282667, PMID: 35210789 PMC8863331

[12] Kadel S Ainsua-Enrich E Hatipoglu I Turner S Singh S Khan S. A major population of functional KLRG1(-) ILC2s in female lungs contributes to a sex bias in ILC2 numbers. Immunohorizons. (2018) 2:74–86. doi: 10.4049/immunohorizons.1800008, PMID: 29568816 PMC5860819

[13] Trivedi S Labuz D Deering-Rice CE Kim CU Christensen H Aamodt S. IL-33 induces NF-kappaB activation in ILC2 that can be suppressed by *in vivo* and ex vivo 17beta-estradiol. Front Allergy. (2022) 3:1062412. doi: 10.3389/falgy.2022.1062412, PMID: 36506643 PMC9732027

[14] Xia J Zhao J Shang J Li M Zeng Z Zhao J. Increased IL-33 expression in chronic obstructive pulmonary disease. Am J Physiology: Lung Cell Mol Physiol. (2015) 308:L619–627. doi: 10.1152/ajplung.00305.2014, PMID: 25595648

[15] Wu W Xu Y He X Lu Y Guo Y Yin Z. IL-33 promotes mouse keratinocyte-derived chemokine, an IL-8 homologue, expression in airway smooth muscle cells in ovalbumin-sensitized mice. Asian Pacific J Allergy Immunol. (2014) 32:337–44. doi: 10.1152/ajplung.00305.2014, PMID: 25543045

[16] Warren KJ Poole JA Sweeter JM DeVasure JM Dickinson JD Peebles RS Jr. Neutralization of IL-33 modifies the type 2 and type 3 inflammatory signature of viral induced asthma exacerbation. Respir Res. (2021) 22:206. doi: 10.1186/s12931-021-01799-5, PMID: 34266437 PMC8281667

[17] Gaurav R Poole JA. Interleukin (IL)-33 immunobiology in asthma and airway inflammatory diseases. J asthma: Off J Assoc Care Asthma. (2022) 59:2530–8. doi: 10.1080/02770903.2021.2020815, PMID: 34928757 PMC9234100

[18] Ong CB Kumagai K Brooks PT Brandenberger C Lewandowski RP Jackson-Humbles DN. Ozone-induced type 2 immunity in nasal airways. Development and lymphoid cell dependence in mice. Am J Respir Cell Mol Biol. (2016) 54:331–40. doi: 10.1165/rcmb.2015-0165OC, PMID: 26203683

[19] Kumagai K Lewandowski R Jackson-Humbles DN Li N Van Dyken SJ Wagner JG. Ozone-induced nasal type 2 immunity in mice is dependent on innate lymphoid cells. Am J Respir Cell Mol Biol. (2016) 54:782–91. doi: 10.1165/rcmb.2015-0118OC, PMID: 26559808

[20] Gandhi NA Bennett BL Graham NM Pirozzi G Stahl N Yancopoulos GD. Targeting key proximal drivers of type 2 inflammation in disease. Nat Rev Drug Discov. (2016) 15:35–50. doi: 10.1038/nrd4624, PMID: 26471366

[21] Zhou S Qi F Gong Y Zhang J Zhu B. Biological therapies for atopic dermatitis: A systematic review. Dermatology. (2021) 237:542–52. doi: 10.1159/000514535, PMID: 33735876

[22] Mauvais-Jarvis F Bairey Merz N Barnes PJ Brinton RD Carrero JJ DeMeo DL. Sex and gender: modifiers of health, disease, and medicine. Lancet. (2020) 396:565–82. doi: 10.1016/S0140-6736(20)31561-0, PMID: 32828189 PMC7440877

[23] Townsend EA Miller VM Prakash YS. Sex differences and sex steroids in lung health and disease. Endocrine Rev. (2012) 33:1–47. doi: 10.1210/er.2010-0031, PMID: 22240244 PMC3365843

[24] Zein JG Erzurum SC. Asthma is different in women. Curr Allergy Asthma Rep. (2015) 15:28. doi: 10.1007/s11882-015-0528-y, PMID: 26141573 PMC4572514

[25] Vrieze A Postma DS Kerstjens HA. Perimenstrual asthma: a syndrome without known cause or cure. J Allergy Clin Immunol. (2003) 112:271–82. doi: 10.1067/mai.2003.1676, PMID: 12897732

[26] Murphy VE Gibson PG Schatz M. Managing asthma during pregnancy and the postpartum period. J Allergy Clin Immunol Pract. (2023) 11:3585–94. doi: 10.1016/j.jaip.2023.07.020, PMID: 37482082

[27] Kalidhindi RSR Ambhore NS Balraj P Schmidt T Khan MN Sathish V. Androgen receptor activation alleviates airway hyperresponsiveness, inflammation, and remodeling in a murine model of asthma. Am J Physiology: Lung Cell Mol Physiol. (2021) 320:L803–18. doi: 10.1152/ajplung.00441.2020, PMID: 33719566 PMC8174830

[28] Chowdhury NU Cephus JY Henriquez Pilier E Wolf MM Madden MZ Kuehnle SN. Androgen signaling restricts glutaminolysis to drive sex-specific Th17 metabolism in allergic airway inflammation. J Clin Invest. (2024) 134(23):e177242. doi: 10.1172/JCI177242, PMID: 39404231 PMC11601904

[29] Zhao Y Zhu Y Chen X Lin H Qin N Zhou Z. Circulating innate lymphoid cells exhibit distinctive distribution during normal pregnancy. Reprod Sci. (2022) 29(4):1124–35. doi: 10.1007/s43032-021-00834-6, PMID: 34988918 PMC8907087

[30] Ambhore NS Kalidhindi RSR Loganathan J Sathish V. Role of differential estrogen receptor activation in airway hyperreactivity and remodeling in a murine model of asthma. Am J Respir Cell Mol Biol. (2019) 61:469–80. doi: 10.1165/rcmb.2018-0321OC, PMID: 30958966 PMC6775953

[31] Kalidhindi RSR Ambhore NS Bhallamudi S Loganathan J Sathish V. Role of Estrogen Receptors alpha and beta in a Murine Model of Asthma: Exacerbated Airway Hyperresponsiveness and Remodeling in ERbeta Knockout Mice. Front Pharmacol. (2019) 10:1499. doi: 10.3389/fphar.2019.01499, PMID: 32116656 PMC7010956

[32] Itoga M Ishioka Y Makiguchi T Tanaka H Taima K Saito N. Role of G-protein-coupled estrogen receptor in the pathogenesis of chronic asthma. Immunol Lett. (2024) 265:16–22. doi: 10.1016/j.imlet.2023.12.001, PMID: 38142780

[33] Warren KJ Deering-Rice C Huecksteadt T Trivedi S Venosa A Reilly C. Steady-state estradiol triggers a unique innate immune response to allergen resulting in increased airway resistance. Biol Sex Differences. (2023) 14(1):2. doi: 10.1186/s13293-022-00483-7, PMID: 36609358 PMC9817388

[34] Reilly CA Crouch DJ. Analysis of the nutritional supplement 1AD, its metabolites, and related endogenous hormones in biological matrices using liquid chromatography-tandem mass spectrometry. J Anal Toxicol. (2004) 28:1–10. doi: 10.1093/jat/28.1.1, PMID: 14987417

[35] Warren KJ Sweeter JM Pavlik JA Nelson AJ Devasure JM Dickinson JD. Sex differences in activation of lung-related type 2 innate lymphoid cells in experimental asthma. Ann Allergy Asthma Immunology: Off Publ Am Coll Allergy Asthma Immunol. (2017) 118(2):233–4. doi: 10.1016/j.anai.2016.11.011, PMID: 28017508 PMC5291757

[36] van den Berge M Heijink HI van Oosterhout AJ Postma DS. The role of female sex hormones in the development and severity of allergic and non-allergic asthma. Clin Exp Allergy: J Br Soc Allergy Clin Immunol. (2009) 39:1477–81. doi: 10.1111/j.1365-2222.2009.03354.x, PMID: 19954427

[37] Trivedi S Deering-Rice CE Aamodt SE Huecksteadt TP Myers EJ Sanders KA. Progesterone amplifies allergic inflammation and airway pathology in association with higher lung ILC2 responses. Am J Physiology: Lung Cell Mol Physiol. (2024) 327(1):L65–78. doi: 10.1152/ajplung.00207.2023, PMID: 38651968 PMC11380947

[38] Warren KJ Fang X Gowda NM Thompson JJ Heller NM. The TORC1-activated proteins, P70S6K and GRB10, regulate IL-4 signaling and M2 macrophage polarization by modulating phosphorylation of insulin receptor substrate-2. J Biol Chem. (2016) 291(48):24922–30. doi: 10.1074/jbc.M116.756791, PMID: 27742835 PMC5122764

[39] Cephus JY Gandhi VD Shah R Brooke Davis J Fuseini H Yung JA. Estrogen receptor-alpha signaling increases allergen-induced IL-33 release and airway inflammation. Allergy. (2020) 76(1):255–68. doi: 10.1111/all.14491, PMID: 32648964 PMC7790897

[40] Stier MT Zhang J Goleniewska K Cephus JY Rusznak M Wu L. IL-33 promotes the egress of group 2 innate lymphoid cells from the bone marrow. J Exp Med. (2018) 215(1):263–81. doi: 10.1084/jem.20170449, PMID: 29222107 PMC5748848

