# Supplementary material for: Serum 17β-estradiol inversely correlates with circulating group 2 innate lymphoid cells in a cohort of asthmatic patients
Source: Front Immunol. 2025 Aug 1;16:1555228. doi: 10.3389/fimmu.2025.1555228 (PMC12354583; doi:10.3389/fimmu.2025.1555228)
Supplement: Supplementary file 13 [file DataSheet1.pdf]

SUPPLEMENTARY MATERIALS

Supplemental Figures

Figure S1. fMLP-induced calcium response in CTL and *plcg2*^{kd} cells. Calcium responses in the individual CTL (**A**) and *plcg2*^{kd} (**B**) cells in response to fMLP stimulation. See supplementary Video 8. Calcium response is visualized by the fluorescent calcium indicator Fluo-4. Cells were stained with Fluo-4 (green) 30 min prior to the experiment. CTL and *plcg2*^{kd} cells were stimulated with fMLP at a final concentration of 10 nM, 1 nM, or 0.1 nM at time 0 s, respectively. The numbers in **A** and **B** indicate an individual cell.

Figure S2. Comparison of peak PIP_3 response in CTL and *plcg2*^{kd} cells. Peak PH-GFP membrane translocation in the CTL and plcg2kd cells were measured. Mean Mean ± SD is shown. N = 3 in each group of cells. A student's *t*-test was used to calculate the *p*-values. Statistical significance is indicated as follows: *ns* (not significant $p > 0.05$), * ($p < 0.05$), ** ($p < 0.01$).

Supplementary videos:

Video S1-S3. Calcium response in the control (CTL, top) and *plcg2*^{kd} (bottom) human neutrophil-like cell HL60 upon 10 nM (**S1**), 1 nM (**S2**), or 0.1 nM (**S3**) fMLP, respectively. Cells were stained with the calcium indicator Fluo-4 and stimulated with 10, 1, or 0.1 nM fMLP at the beginning of the movies, respectively. Scale bar = 50 μm.

Video S4-S5. Membrane translocation of CAPRI-GFP in the control (CTL, top) and *plcg2*^{kd} (bottom) HL60 cells upon 10 nM (**S4**) or 0.1 nM (**S5**) fMLP, respectively. Cells expressing CAPRI-GFP (green) were stimulated with 10, 1, or 0.1 nM fMLP at the beginning of the movies, respectively. To visualize the application of the stimuli, fMLP was mixed with a red fluorescent dye Alexa 594 (red). Scale bar = 5 μm.

Video S6-S7. Membrane translocation of active Ras biosensor, RBD-RFP, in the control (CTL, top) and *plcg2*^{kd} (bottom) HL60 cells upon fMLP stimulation. Cells expressing RBD-RFP (red) were stimulated with 10 nM (**S7**) or 0.1 nM (**S8**) fMLP at the beginning of the movies. To visualize the application of the stimuli, fMLP was mixed with a green, fluorescent dye Alexa 488 (green). Scale bar = 5 μm.

Video S8-S9. Membrane translocation of PIP_3 biosensor, PH-GFP, in the control (CTL, top) and *plcg2*^{kd} (bottom) HL60 cells upon fMLP stimulation. Cells expressing PH-GFP (green) and a PM marker (red) were stimulated with 10 nM (**S9**) or 0.1 nM (**S10**) fMLP, respectively, at the beginning of the movies. Scale bar = 5 μm.

Video S10-11. Monitoring fMLP-induced actin polymerization in CTL (top) and *plcg2*^{kd} (bottom) cells upon fMLP stimulation. Actin polymerization is monitored by the membrane translocation of GFP-F-tractin in cells. 10 nM (**S11**) or 0.1 nM nM (**S12**) fMLP was added to the cells at the beginning of the movies. Scale bar = 5 μm.

Video S12. Montaged movie showing the migration behavior of CTL (left) and *plcg2kd* (right) cells under different conditions. Cells were exposed to either no gradient (top row) or to chemoattractant gradients generated from localized sources of fMLP (top two pairs), SDF-1α (middle two pairs), or LTB4 (bottom two pairs). For each chemoattractant, the upper panel represents a gradient of 0.1 nM, and the lower panel represents a gradient of 100 nM.

Xu et al., Figure S1

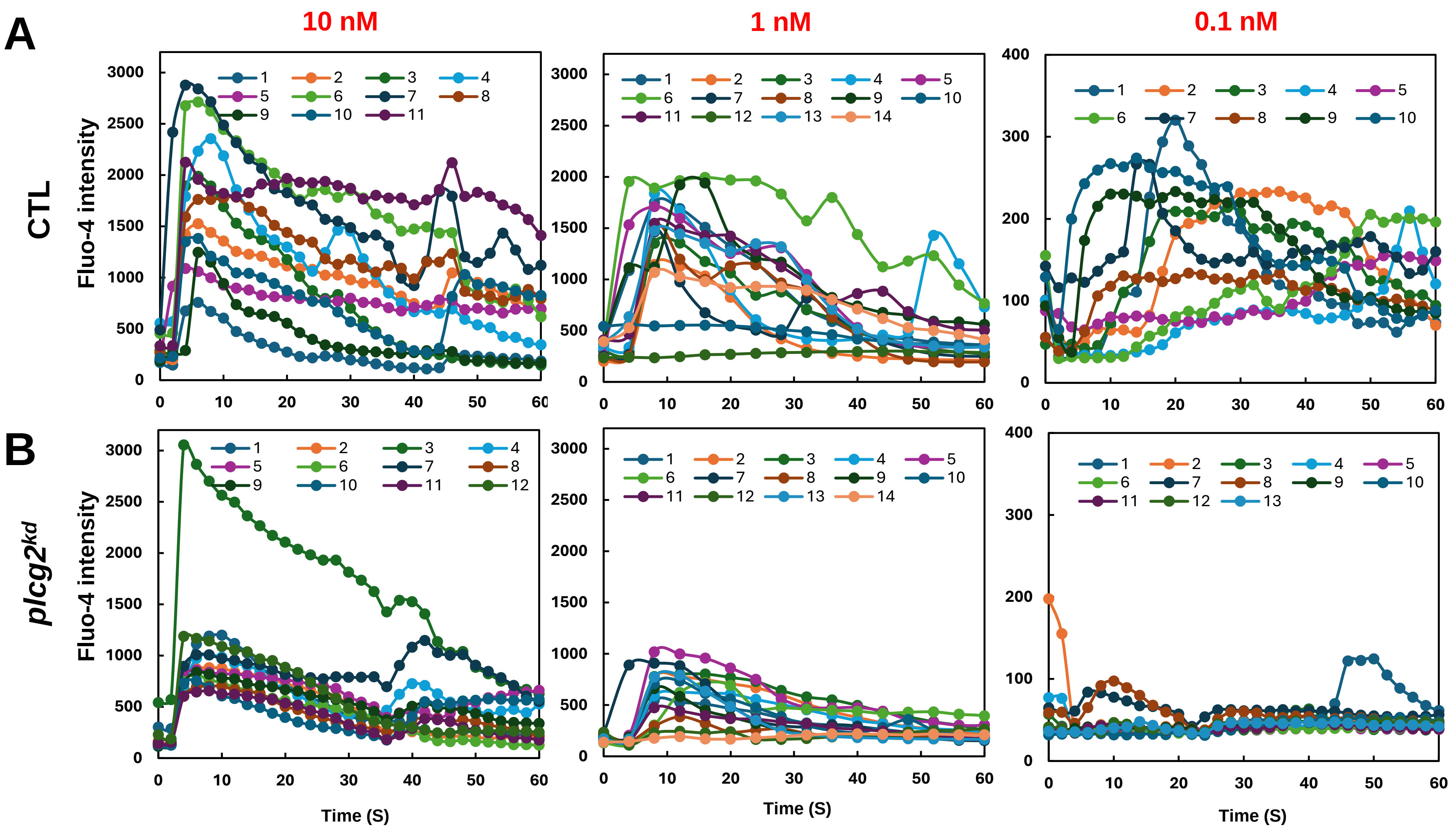

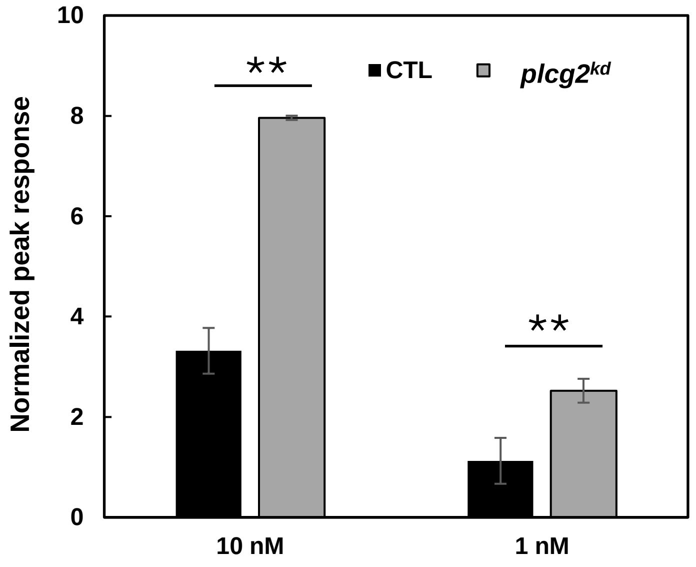
10
8
6
4
2
0
Normalized peak response
**
CTL
plcg2kd
**
10 nM
1 nM
